# Fast assessment of long axis strain with standard cardiovascular magnetic resonance: a validation study of a novel parameter with reference values

**DOI:** 10.1186/s12968-015-0171-8

**Published:** 2015-08-08

**Authors:** Johannes H. Riffel, Florian Andre, Malte Maertens, Franziska Rost, Marius G. P. Keller, Sorin Giusca, Sebastian Seitz, Arnt V. Kristen, Matthias Müller, Evangelos Giannitsis, Grigorios Korosoglou, Hugo A. Katus, Sebastian J. Buss

**Affiliations:** Department of Cardiology, Angiology and Pneumology, University of Heidelberg, INF 410, 69120 Heidelberg, Germany; DZHK (German Centre for Cardiovascular Research), Heidelberg, Germany

**Keywords:** Cardiovascular magnetic resonance, Cardiomyopathy, Long axis function, Strain

## Abstract

**Background:**

Assessment of longitudinal function with cardiovascular magnetic resonance (CMR) is limited to measurement of systolic excursion of the mitral annulus (MAPSE) or elaborate strain imaging modalities. The aim of this study was to develop a fast assessable parameter for the measurement of long axis strain (LAS) with CMR.

**Methods:**

40 healthy volunteers and 125 patients with different forms of cardiomyopathy were retrospectively analyzed. Four different approaches for the assessment of LAS with CMR measuring the distance between the LV apex and a line connecting the origins of the mitral valve leaflets in enddiastole and endsystole were evaluated. Values for LAS were calculated according to the strain formula.

**Results:**

LAS derived from the distance of the epicardial apical border to the midpoint of the line connecting the mitral valve insertion points (LAS-epi/mid) proved to be the most reliable parameter for the assessment of LAS among the different approaches.

LAS-epi/mid displayed the highest sensitivity (81.6 %) and specificity (97.5 %), furthermore showing the best correlation with feature tracking (FTI) derived transmural longitudinal strain (r = 0.85). Moreover, LAS-epi/mid was non-inferior to FTI in discriminating controls from patients (Area under the curve (AUC) = 0.95 vs. 0.94, p = NS). The time required for analysis of LAS-epi/mid was significantly shorter than for FTI (67 ± 8 s vs. 180 ± 14 s, p < 0.0001). Additionally, LAS-epi/mid performed significantly better than MAPSE (Delta AUC = 0.09; p < 0.005) and the ejection fraction (Delta AUC = 0.11; p = 0.0002).

Reference values were derived from 234 selected healthy volunteers. Mean value for LAS-epi/mid was −17.1 ± 2.3 %. Mean values for men were significantly lower compared to women (−16.5 ± 2.2 vs. -17.9 ± 2.1 %; p < 0.0001), while LAS decreased with age.

**Conclusions:**

LAS-epi/mid is a novel and fast assessable parameter for the analysis of global longitudinal function with non-inferiority compared to transmural longitudinal strain.

## Background

Reduced left ventricular (LV) ejection fraction (EF) is an important predictor for cardiac mortality and morbidity [1]. Accordingly, in clinical routine therapeutic decision-making is still mainly based on the EF [2, 3].

However, precise measurement of LV long axis function may better reflect the contractile function of the myocardium than the geometric analysis of the left ventricle, which is used for the calculation of the EF [4]. Previously, echocardiographic data provided evidence that mitral annular displacement in the longitudinal direction constitutes an important component of the global function of the heart [5–7] and is reduced early in the progression of several pathological conditions [8, 9].

Impairment of LV longitudinal function is recognized as an independent predictor for survival in patients with chronic heart failure [10] and various cardiomyopathies [11–13].

Cardiovascular magnetic resonance (CMR) is nowadays considered as the non-invasive gold standard for the evaluation of LV function [14]. Due to its excellent intrinsic blood-to-tissue contrast, epicardial and endocardial contours can be detected more precisely in CMR than with echocardiography. In CMR, atrioventricular plane displacement has been identified as the major contributor to stroke volume and was best associated with LV function [5]. In clinicial routine, the only indicator of longitudinal function is the measurement of systolic excursion of the mitral annulus (MAPSE).

Evaluation of longitudinal deformation with different software packages is still time consuming and, more importantly, standardization of strain imaging for better comparability is urgently needed. The aim of our study was to evaluate long axis strain (LAS) with CMR as a rapid assessable and feasible parameter for the determination of LV longitudinal function.

## Methods

### Study population

The study population consisted of 125 patients with a clinical indication for CMR and 40 healthy volunteers who were analyzed retrospectively. 60 patients had dilated cardiomyopathy (DCM), 25 patients were diagnosed with hypertrophic cardiomyopathy (HCM) and 40 patients had biopsy-confirmed systemic light-chain (AL) amyloidosis. All patients were in clinical stable condition (NYHA ≤ III) and showed sinus rhythm during the examination. For the assessment of reference values 234 healthy volunteers (101 women and 133 men) were analyzed. The investigation was carried out after approval by the local Ethics Committee of the University of Heidelberg and in accordance with the Declaration of Helsinki.

### CMR acquisition protocol and CMR analysis (standard parameters)

CMR was performed on a 1.5 T clinical scanner (Achieva, Philips Healthcare, Best, The Netherlands) equipped with a cardiac phased array 32-channel receiver coil. Cine images were obtained using a breath-hold segmented-k-space balanced fast-field echo sequence (SSFP) employing retrospective ECG gating in long axis planes (2, 3 and 4 chamber views) as well as in contiguous short axis slices (2 mm gap) covering the whole ventricles from the annulus of the AV valves to the apex, with 35 phases per cardiac cycle. Typical CMR imaging parameters were: field-of-view (FOV) = 350 × 350 mm^2^, repetition time/echo time (TR/TE) = 2.8/1.4 ms, acquired voxel size = 2.2 × 2.2 × 8 mm^3^, flip angle (FA) = 60°, reconstructed voxel size = 1.3 × 1.2 × 8 mm^3^).

All analyses were performed on a commercially available workstation (IntelliSpace Portal (ISP) Version 6, Philips Healthcare, Best, The Netherlands) equipped with a semi-automatic software for volumetric analysis. Results for ventricular volumes, ejection fraction and LV myocardial mass were derived from short axis slices after manually tracing of epi- and endocardial borders, excluding papillary muscles from the myocardium.

### Feature tracking imaging (FTI)

For strain analysis long axis views of the ventricle in 2- and 4 chamber views were divided according to the standardized 17 segment model. The apical cap (segment 17) and the segments of the 3 chamber view were not considered for analysis. Retrospective image analyses were conducted employing the 2D CPA CMR Feature tracking software (TomTec Imaging Systems, Munich, Germany). This tool comprised a software based feature tracking algorithm, which has been validated previously in experimental and clinical studies [15–17]. Feature tracking measures longitudinal strain and strain rate as well as myocardial velocities along a user-defined endocardial and epicardial border throughout the cardiac cycle on standard cine CMR images. Endocardial as well as epicardial borders are initially set in end-diastole of standard cine SSFP long axis images (2 and 4 chamber view). The software algorithm then tracks automatically image features like signal inhomogenities, tissue patterns of the myocardium or anatomic structures throughout the whole cardiac cycle.

In our study global transmural longitudinal strain represents the mean values of epicardial and endocardial strain and was assessed by measuring the average peak of the mean curve of all segments. The curve represents the average of all segments over the whole cardiac cycle. This value was measured three times and then averaged, resulting in the mean transmural longitudinal strain. The technique has been described in detail in a recently published study [18].

### Long axis strain (LAS)

Long axis strain was assessed in 2 and 4 chamber views by measuring displacement of the mitral annulus. We evaluated and compared 4 different approaches for measuring LAS. All values were assessed with the IntelliSpace Portal (ISP) workspace (Version 6, Philips Healthcare, Best, The Netherlands).

**Fig. 1:**
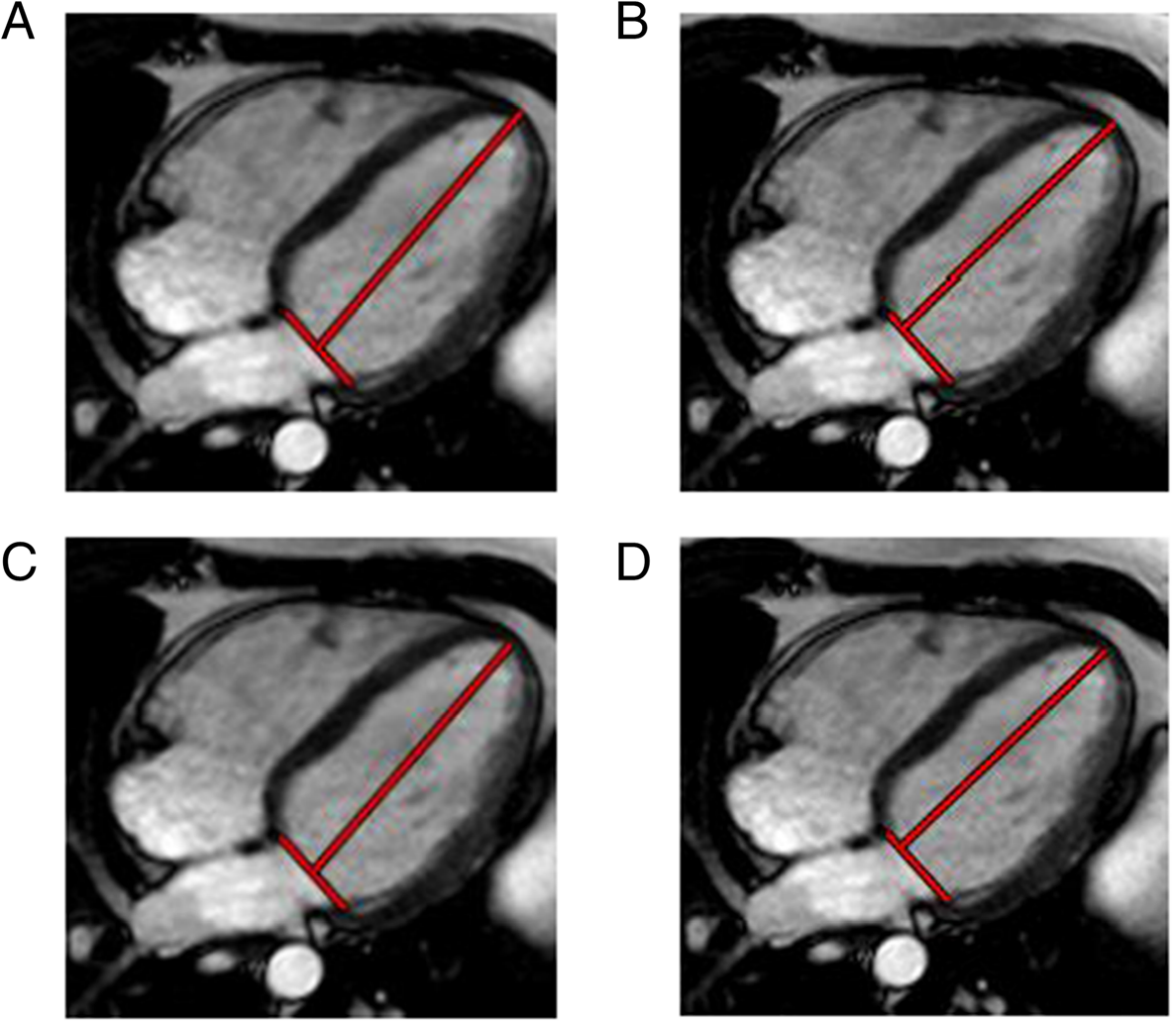
**a**-**d** Representative images illustrating the different techniques for assessment of long axis strain (LAS) in a control subject. Shown are the measurements in enddiastole

LAS-epi/mid: The distance between the epicardial border of the LV apex and the middle of a line connecting the origins of the mitral valve leaflets was measured in both endsystole and enddiastole (Fig. 1a).

LAS-endo/mid: The distance between the endocardium of the LV apex and the middle of a line connecting the origins of the mitral valve leaflets was measured in endsystole and enddiastole, respectively (Fig. 1b).

LAS-epi/perp.: The distance between the epicardal border of the LV apex and a line connecting the origins of the mitral valve leaflets was measured perpendicularly in endsystole as well as in enddiastole (Fig. 1c).

LAS-endo/perp.: The distance between the endocardium of the LV apex and a line connecting the origins of the mitral valve leaflets was measured perpendicularly in endsystole and in enddiastole, respectively (Fig. 1d).

The value in percentage for LAS was finally measured according to the strain formula:

$$ LAS=\frac{lengt{h}_{endsystole}\kern0.75em -\kern0.5em  lengt{h}_{enddiastole}}{lengt{h}_{enddiastole}}*100 $$

Mean values in 2 and 4 chamber views were calculated. The mean of three measurements was then used for further analysis.

### Intra- and interobserver variability and time requirements

CMR examinations were analyzed by the investigators in a blinded manner. For calculation of intra- and interobserver variability 20 single measurements were used. To examine intraobserver variability, a sample of 20 randomly selected CMR examinations for the measurement of LAS were randomly selected for masked review by the same investigator. The same studies were analysed by a co-investigator, who was blinded to the clinical information and the results of the first investigation, in order to measure interobserver variability.

The required time for LAS analysis was registered in another 20 CMR datasets.

### Statistical analysis

Data are presented as mean ± standard deviation. Differences in values for LAS were evaluated using unpaired Student’s t-test with Welch correction for samples with potentially unequal variances or one-way ANOVA with Dunnett’s post-hoc test when appropriate. Pearson’s correlation coefficients were calculated since all variables showed normal distribution according to D’Agostino Pearson test. Linear regression analysis was used to model the relationship between LAS and transmural longitudinal strain. Receiver operator characteristic (ROC) curves were used to determine the optimal prognostic LAS cut-off value (highest sum of sensitivity and specificity) for discriminating healthy controls and patients. For the assessment of intra- and interobserver agreement variation coefficients were calculated. A p-value <0.05 was considered as statistically significant. All Statistics were calculated using MedCalc 12.3 (MedCalc software, Mariakerke, Belgium).

## Results

### Statistical data of study population

125 patients and 40 healthy subjects were retrospectively analyzed. Mean age was 55 ± 14 years and 52 ± 12 years (p = 0.15), respectively. EF was significantly lower in patients with cardiomyopathies (46 ± 17 % vs. 65 ± 6; p < 0.0001). Mean value for body mass index (BMI) was 25 ± 3 in healthy controls and 26 ± 4 in patients (p = 0.06).

Moreover 234 healthy volunteers were analyzed for the assessment of reference values. A detailed characteristic of these subjects is shown in Table 1.

**Table 1:** Characteristics of the study population (n = 234) for assessment of reference values of LAS-epi/mid (BMI = body mass index, EF = ejection fraction)

Parameter	Result
Age (years)	48 ± 13
Gender	
Male	133 (56.8 %)
Female	101 (43.2 %)
BMI (Kg/m^2^)	25 ± 3
EF (%)	63 ± 5

### Long axis strain (LAS)

**Fig. 2:**
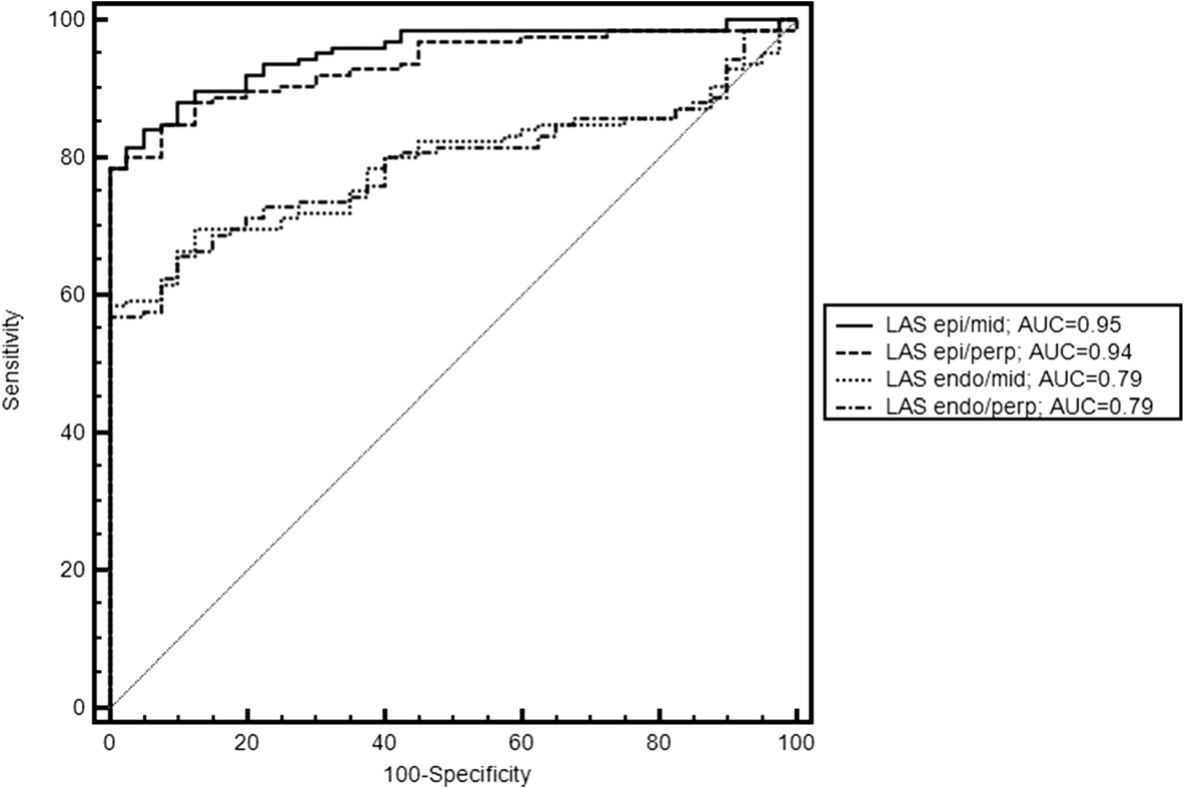
ROC analysis of the 4 different approaches for the assessment of LAS. LAS-epi/mid and LAS-epi/perp performed significantly better in discriminating healthy subjects from patients than LAS-endo/mid and LAS-endo/perp, respectively. Values for area under the curve (AUC) were significantly higher in LAS-epi/mid and LAS epi/perp (0.95 and 0.93 vs. 0.79 and 0.79 p < 0.0001)

**Fig. 3:**
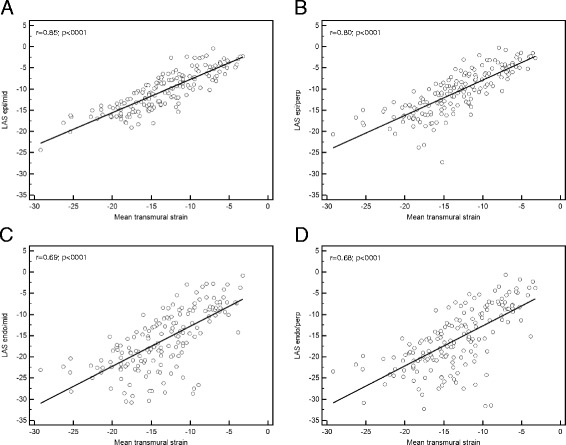
**a**-**d** Regression lines of the 4 different approaches for measurement of LAS compared with FTI derived transmural strain. LAS-epi/mid (r = 0.85) and LAS-epi/perp (r = 0.80) showed the best correlation with transmural strain imaging

**Fig. 4:**
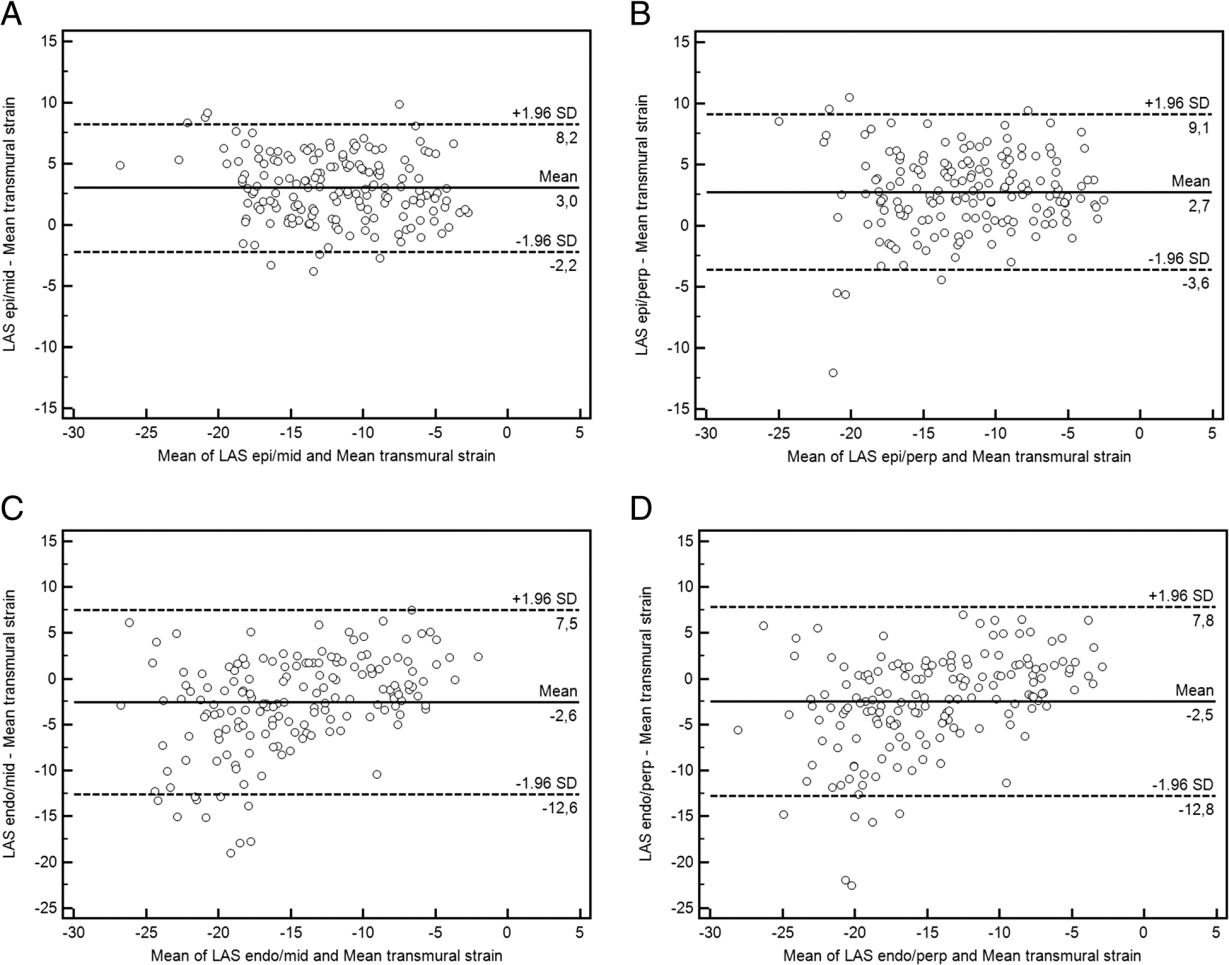
**a**-**d** Bland Altman plots showing comparisons between the 4 different approaches and FTI derived transmural strain. The bias (solid line) and limits of agreement (blue dashed lines) for the 4 methods are shown in each graph

We evaluated 4 different techniques for the assessment of LAS. Mean value in healthy controls was −15.9 ± 2.2 % for LAS-epi/mid, −16.4 ± 2.5 % for LAS-epi/perp, −21.0 ± 3.6 % for LAS endo/mid and −21.1 ± 3.9 % for LAS endo/perp, respectively. Mean values for patients with cardiomyopathies were significantly lower (LAS-epi/mid:-8.7 ± 3.8 %, p < 0.0001; LAS-epi/perp: −8.9 ± 4.6 %, p < 0.0001, LAS-endo/mid: −14.4 ± 7.1 %, p < 0.0001 and LAS endo/perp −14.2 ± 7.1 %, p < 0.0001). Mean values for mean transmural strain were −19.3 ± 3.6 % in controls and −11.6 ± 4.0 % (p < 0.0001) in patients, respectively. Detailed information about the mean values of the different cardiomyopathies is shown in Table 2 .For discrimination of healthy controls and patients, LAS–epi/mid and LAS-epi/perp provided the highest sensitivity and specificity (81.6 % and 97.5 % vs. 78.4 % and 100 %) among the 4 methods. The area under the ROC curve (AUC) was 0.95 for LAS-epi/mid and 0.94 for LAS-epi/perp, respectively. Values for AUC were significantly lower for LAS-endo/mid (0.79, p < 0.0001) and LAS-endo/perp (0.79, p < 0.0001) (Fig 2). LAS-epi/mid showed the strongest correlation with mean longitudinal strain (r = 0.85) with a small bias (+3.0 %) and narrow 95 % limits of agreement (LOA, ±5.2 %). Correlation of LAS- epi/perp was also strong (r = 0.80), while correlation was weaker for LAS-endo/mid (r = 0.69) and LAS-endo/perp (r = 0.68). The corresponding regression lines and Bland-Altman plots are shown in Figs. 3 and 4. Comparison of the 4 different techniques identified LAS-epi/mid as the most significant parameter for the assessment of LAS with the best correlation with FTI derived transmural longitudinal strain and highest sensitivity and specificity.

**Table 2:** Mean values for the different techniques of LAS and transmural longitudinal strain of the study population

	Healthy volunteers (n = 40)	Patients (n = 125)	AL-amyloidosis (n = 40)	DCM (n = 60)	HCM (n = 25)
LAS-epi/mid (%)	−15.9 ± 2.2	−8.7 ± 3.8*	−8.0 ± 3.4*	−8.1 ± 3.8*	−11.4 ± 3.5*
LAS-epi/perp (%)	−16.4 ± 2.5	−8.9 ± 4.6*	−7.8 ± 3.6*	−7.8 ± 4.0*	−13.1 ± 4.8*
LAS-endo/mid (%)	−21.0 ± 3.6	−14.4 ± 7.1*	−16.2 ± 6.0*	−10.1 ± 4.6*	−22.1 ± 6.1
LAS-endo/perp (%)	−21.1 ± 3.9	−14.2 ± 7.1*	−16.4 ± 6.7*	−10.0 ± 4.9*	−20.9 ± 5.7
Mean transmural strain (%)	−19.3 ± 3.6	−11.6 ± 4.0*	−11.6 ± 4.3*	−10.5 ± 3.7*	−14.0 ± 2.9*

* = significant difference between patients and controls

### Prediction of structural heart disease and comparison with FTI derived transmural longitudinal strain imaging

The cut-off value to discriminate healthy subjects from patients with different cardiomyopathies with the highest sensitivity and specificity was −12.4 % for LAS-epi/mid and −15.3 % for transmural longitudinal strain, respectively. Compared to strain imaging, LAS-epi/mid showed similar values for sensitivity (81.6 % vs. 84.0 %) and specificity (97.5 % vs. 92.5 %). ROC analysis revealed a comparable AUC for LAS-epi/mid (0.95) and transmural longitudinal strain (0.94, p = NS), respectively.

### Comparison of LAS with standard parameters

**Fig. 5:**
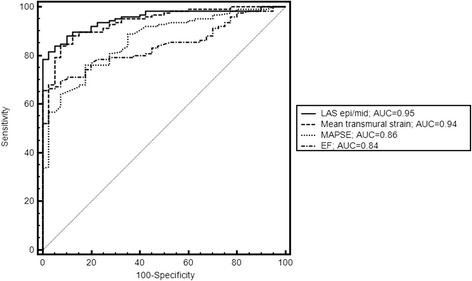
ROC analysis showed comparable values for AUC for LAS-epi/mid and transmural longitudinal strain. Values for MAPSE and the EF were significantly lower. The cut off value for LA-epi/mid for discriminating controls and patients was −12.4 %

LAS-epi/mid performed significantly better than other parameters of cardiac function such as MAPSE (Delta AUC = 0.09; p < 0.005) and the EF (Delta AUC = 0.11; p = 0.0002) in discriminating patients from healthy subjects. The corresponding ROC analysis is shown in Fig. 5.

### Intra- and interobserver variability and time requirements

Intra- and interobserver variability for single measurements of LAS-epi/mid were low with 5.6 ± 4.2 % and 6.3 ± 4.2 %, respectively. Intra- and interobserver variability for LAS-epi/perp were −6.1 ± 8.1 % and −6.5 ± 6.1 %, for LAS-endo/mid −7.2 ± 7.0 % and −6.1 ± 7.2 % and for LAS-endo/perp −6.0 ± 5.8 % and 5.9 ± 5.9 %.

The time required for the measurement of LAS-epi/mid in 4 chamber view and 2 chamber view was 67 ± 8 s and significantly shorter than the measurement time for FTI derived transmural longitudinal strain analysis, which was also assessed in 2- and 4 chamber view (180 ± 14 s, p < 0.0001).

### Reference values and age- and gender-related differences

**Fig. 6:**
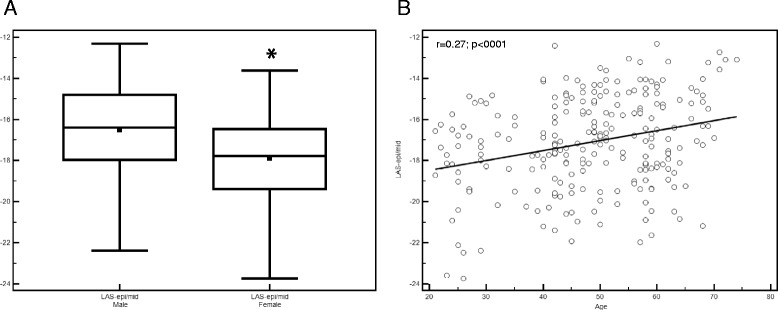
**a** and **b** Box and Whiskers plot of LAS-epi/mid for the comparison of male and female showed significant higher values in male subjects (* = significant difference between male and female, p < 0.0001). Regression analysis revealed significant lower values for LAS-epi/mid in younger persons (p < 0.001)

Reference values were derived from 234 healthy volunteers, the resulting mean value for LAS-epi/mid was −17.1 ± 2.3 %. Additionally, LAS-epi/mid was analyzed related to age and gender. Mean values for men were significantly lower compared to women (−16.5 ± 2.2 vs. -17.9 ± 2.1 %; p < 0.0001) (Fig 6a) and decreased significantly with age (p < 0.001) (Fig 6b).

## Discussion

In our study, we demonstrated the feasibility of a novel and readily available parameter for the measurement of global longitudinal LV function using standard CMR images. LAS-epi/mid correlated strongly with FTI derived transmural longitudinal strain, while sensitivity as well as specificity data were comparable. LAS-epi/mid performed significantly better in discriminating healthy subjects from patients with cardiomyopathies than EF and MAPSE. Additionally, we provided reference values for LAS-epi/mid. Atrioventricular plane displacement, a measure for longitudinal function, is known to provide approximately 60 % of the total LV stroke volume [5]. Different studies have shown that longitudinal function also serves as an early and independent predictor for the outcome in several cardiac diseases and cardiomyopathies [19–22]. However, assessment of longitudinal function in CMR is still limited to measurement of MAPSE or elaborate strain imaging modalities. Until now there are no data about assessment of MAPSE in CMR regarding correlation with strain imaging or prognosis in different cardiomyopathies. An additional drawback of MAPSE is that only absolute but not relative values are measured, which means that the length of the LV is not considered in the evaluation of longitudinal function. Nevertheless, MAPSE can be measured rapidly from normal cine sequences and is therefore a suitable parameter for longitudinal function in clinical practice. For the quantification of longitudinal function with strain imaging, there are several software packages and analysis tools available, which are mainly designed for the offline analysis and often need additional or special pulse sequences. While feature tracking analysis can be performed offline with normal cine sequences with a special software tool, for tagging or SENC analysis a gradient echo-based tagging pulse sequence is required [17, 23, 24]. There are different CMR studies about longitudinal function assessed with strain imaging techniques. A study of Neizel et al. could show that objective quantification of regional myocardial function with SENC could discriminate between different transmurality levels in patients with acute myocardial infarction [25]. Moreover, with SENC significant coronary artery disease could already be detected during intermediate stress with similar accuracy compared to standard peak stress cine sequences [26] . Recently, we showed in a prospective CMR study with 210 DCM patients that the assessment of LV longitudinal function with FTI serves as an independent predictor of survival [18]. In addition, in a study of Miszalski-Jamka et al. patients with Churgh-Strauss-Syndrom and Wegener's granumolatosis displayed reduced values for longitudinal strain measured with feature tracking software despite clinical remission, normal ECG and normal echocardiography [27]. However, in a validation study of the feature tracking algorithm, Augustine et al. showed that feature tracking of circumferential strain displayed reasonable agreement with tagging and acceptable inter-observer reproducibility, but comparability and reproducibility with longitudinal strain was poor [28]. Still, a standardized and reproducible approach for longitudinal strain imaging is not available for CMR which hinders its implementation in clinical routine analysis and reporting. Saba et al. took a different approach to evaluate longitudinal function. In their study the longitudinal motion of the lateral and septal atrioventricular junction was measured using a special tracking software algorithm. The values assessed with this novel method for measurement of mitral annular motion were significantly different in HCM patients compared to healthy volunteers. The measurement time in this study was approximately 10 minutes per subject [29]. Similar to strain imaging this analysis required a special software tool for offline analysis. Bonnemains et al. on the other hand validated a surface–length index as a novel parameter of longitudinal function of the right ventricle [30] and observed that this index allows a rapid detection of abnormal RV-EF during CMR. In a recently published study, Gjesdal et al. analyzed LAS by measuring the distances from the mitral valve insertions to the epicardial apex, which is a more similar approach compared to our technique. They observed a good correlation between LAS assessed with echocardiography and CMR and infarct mass in patients with former myocardial infarction [31]. In a previous echocardiographic study of the same group, Gjesdal et al. could show that assessment of mitral annular plane displacement assessed in a 6-segment LV model did not lead to an improvement of the correlation to infarct mass compared with the 4-segment model. The authors therefore conclude that the commonly used 4-segment model assessed with 2- and 4-chamber view is a reasonable approach for clinical practice [32]. This is in line with our own findings, as addition of 3-chamber view did not lead to better correlation of LAS with FTI derived strain (data not shown).

In our study we wanted to evaluate an easily reproducible and more importantly fast assessable parameter for global longitudinal function, which then can be implemented into clinical routine. A practical advantage of our technique compared to various other strain imaging modalities is that no special CMR sequences or additional software tools are needed. LAS can be assessed online during a standard CMR protocol from standard SSFP sequences. Furthermore, the time required for analysis was much shorter than measurement of transmural strain. Besides, as we could prove that single measurements of LAS showed good intra- and interobserver variability an averaged value for LAS of repeated measurements is redundant.

A slight drawback of our technique compared to feature tracking imaging or the technique applied by Saba et al. is that it does not provide information about strain rate or circumferential and radial strain, which may in certain circumstances reflect cardiac function better [29, 33].

However, since LAS showed a high sensitivity and specificity in discriminating patients with cardiomyopathies from controls, it may be a rapid and valuable screening tool for patients with cardiomyopathies in clinical routine.

## Limitations

The investigation was carried out retrospectively. Additionally, comparison and correlation with other imaging or strain modalities were not assessed in our study. Moreover, compared to feature tracking or tagging, LAS offers only global values for longitudinal strain and provides no data about regional deformation.

## Conclusions

LAS-epi/mid is a reliable and fast assessable parameter for analysis of global longitudinal function in CMR, without the need of additional pulse sequences and off-line processing using special software tools. LAS-epi/mid showed a high correlation with feature tracking analysis while being non-inferior to strain imaging in discriminating healthy controls from patients with cardiomyopathies.

## Abbreviations

LAS: Long axis strain
MAPSE: Mitral annular plane systolic excursion
FTI: Feature tracking imaging
AUC: Area under the curve
LV: Left ventricular
EF: Ejection fraction
CMR: Cardiovascular magnetic resonance
LOA: Limits of agreement
ES: Endsystolic
ED: Enddiastolic
SSFP: Steady state free precession
FOV: Field of view
TR: Repetition time
TE: Echo time
FA: Flip angle
SENC: Strain encoded imaging
ISP: IntelliSpace Portal
ROC: Receiver operator characteristic
BMI: Body mass index
RV: Right ventricular
DCM: Dilated cardiomyopathy
HCM: Hypertrophic cardiomyopathy

## References

[1] Pocock SJ, Wang D, Pfeffer MA, Yusuf S, Mcmurray JJ, Swedberg KB, Ostergren J (2006). Predictors of mortality and morbidity in patients with chronic heart failure. EurHeart J.

[2] Moss AJ, Zareba W, Hall WJ, Klein H, Wilber DJ, Cannom DS, Daubert JP (2002). Prophylactic implantation of a defibrillator in patients with myocardial infarction and reduced ejection fraction. N Engl J Med.

[3] Cleland JG, Daubert JC, Erdmann E, Freemantle N, Gras D, Kappenberger L, Tavazzi L (2005). The effect of cardiac resynchronization on morbidity and mortality in heart failure. N Engl J Med.

[4] Dumesnil JG, Shoucri RM, Laurenceau JL, Turcot J (1979). A mathematical model of the dynamic geometry of the intact left ventricle and its application to clinical data. Circulation.

[5] Carlsson M, Ugander M, Mosen H, Buhre T, Arheden H (2007). Atrioventricular plane displacement is the major contributor to left ventricular pumping in healthy adults, athletes, and patients with dilated cardiomyopathy. Am J Physiol Heart Circ Physiol.

[6] Butz T, Piper C, Langer C, Wiemer M, Kottmann T, Meissner A, Plehn G (2010). Diagnostic superiority of a combined assessment of the systolic and early diastolic mitral annular velocities by tissue Doppler imaging for the differentiation of restrictive cardiomyopathy from constrictive pericarditis. Clin Res Cardiol.

[7] Filusch A, Mereles D, Gruenig E, Buss S, Katus HA, Meyer FJ (2010). Strain and strain rate echocardiography for evaluation of right ventricular dysfunction in patients with idiopathic pulmonary arterial hypertension. Clin Res Cardiol.

[8] Abraham TP, Dimaano VL, Liang HY (2007). Role of tissue Doppler and strain echocardiography in current clinical practice. Circulation.

[9] Buss SJ, Wolf D, Korosoglou G, Max R, Weiss CS, Fischer C, Schellberg D (2010). Myocardial left ventricular dysfunction in patients with systemic lupus erythematosus: new insights from tissue Doppler and strain imaging. J Rheumatol.

[10] Svealv BG, Olofsson EL, Andersson B (2008). Ventricular long-axis function is of major importance for long-term survival in patients with heart failure. Heart.

[11] Willenheimer R (1998). Assessment of left ventricular dysfunction and remodeling by determination of atrioventricular plane displacement and simplified echocardiography. Scand Cardiovasc J Suppl.

[12] Buss SJ, Emami M, Mereles D, Korosoglou G, Kristen AV, Voss A, Schellberg D (2012). Longitudinal left ventricular function for prediction of survival in systemic light-chain amyloidosis: incremental value compared with clinical and biochemical markers. J Am Coll Cardiol.

[13] Kalam K, Otahal P, Marwick TH (2014). Prognostic implications of global LV dysfunction: a systematic review and meta-analysis of global longitudinal strain and ejection fraction. Heart.

[14] Karamitsos TD, Francis JM, Myerson S, Selvanayagam JB, Neubauer S (2009). The role of cardiovascular magnetic resonance imaging in heart failure. J Am Coll Cardiol.

[15] Pirat B, Khoury DS, Hartley CJ, Tiller L, Rao L, Schulz DG, Nagueh SF (2008). A novel feature-tracking echocardiographic method for the quantitation of regional myocardial function: validation in an animal model of ischemia-reperfusion. J Am Coll Cardiol.

[16] Korinek J, Wang J, Sengupta PP, Miyazaki C, Kjaergaard J, McMahon E, Abraham TP (2005). Two-dimensional strain--a Doppler-independent ultrasound method for quantitation of regional deformation: validation in vitro and in vivo. J Am Soc Echocardiogr.

[17] Hor KN, Gottliebson WM, Carson C, Wash E, Cnota J, Fleck R, Wansapura J (2010). Comparison of magnetic resonance feature tracking for strain calculation with harmonic phase imaging analysis. JACC Cardiovasc Imaging.

[18] Buss SJ, Breuninger K, Lehrke S, Voss A, Galuschky C, Lossnitzer D, et al. Assessment of myocardial deformation with cardiac magnetic resonance strain imaging improves risk stratification in patients with dilated cardiomyopathy. Eur Heart J Cardiovasc Imaging. 2014. 10.1093/ehjci/jeu181 25246506

[19] Ersboll M, Valeur N, Mogensen UM, Andersen MJ, Moller JE, Velazquez EJ, Hassager C (2013). Prediction of all-cause mortality and heart failure admissions from global left ventricular longitudinal strain in patients with acute myocardial infarction and preserved left ventricular ejection fraction. J Am Coll Cardiol.

[20] Bertini M, Ng AC, Antoni ML, Nucifora G, Ewe SH, Auger D, Marsan NA (2012). Global longitudinal strain predicts long-term survival in patients with chronic ischemic cardiomyopathy. Circ Cardiovasc Imaging.

[21] Willenheimer R, Cline C, Erhardt L, Israelsson B (1997). Left ventricular atrioventricular plane displacement: an echocardiographic technique for rapid assessment of prognosis in heart failure. Heart.

[22] Sanderson JE (2007). Heart failure with a normal ejection fraction. Heart.

[23] Korosoglou G, Lossnitzer D, Schellberg D, Lewien A, Wochele A, Schaeufele T, Neizel M (2009). Strain-encoded cardiac MRI as an adjunct for dobutamine stress testing: incremental value to conventional wall motion analysis. Circ Cardiovasc Imaging.

[24] Keenan NG, Pennell DJ (2007). CMR of ventricular function. Echocardiography.

[25] Neizel M, Lossnitzer D, Korosoglou G, Schaufele T, Peykarjou H, Steen H, Ocklenburg C (2009). Strain-encoded MRI for evaluation of left ventricular function and transmurality in acute myocardial infarction. Circ Cardiovasc Imaging.

[26] Korosoglou G, Lehrke S, Wochele A, Hoerig B, Lossnitzer D, Steen H, Giannitsis E (2010). Strain-encoded CMR for the detection of inducible ischemia during intermediate stress. JACC Cardiovasc Imaging.

[27] Miszalski-Jamka T, Szczeklik W, Sokolowska B, Karwat K, Belzak K, Mazur W, Kereiakes DJ (2013). Standard and feature tracking magnetic resonance evidence of myocardial involvement in Churg-Strauss syndrome and granulomatosis with polyangiitis (Wegener’s) in patients with normal electrocardiograms and transthoracic echocardiography. Int J Cardiovasc Imaging.

[28] Augustine D, Lewandowski AJ, Lazdam M, Rai A, Francis J, Myerson S, Noble A (2013). Global and regional left ventricular myocardial deformation measures by magnetic resonance feature tracking in healthy volunteers: comparison with tagging and relevance of gender. J Cardiovasc Magn Reson.

[29] Saba SG, Chung S, Bhagavatula S, Donnino R, Srichai MB, Saric M, Katz SD (2014). A novel and practical cardiovascular magnetic resonance method to quantify mitral annular excursion and recoil applied to hypertrophic cardiomyopathy. J Cardiovasc Magn Reson.

[30] Bonnemains L, Mandry D, Menini A, Stos B, Felblinger J, Marie PY, Vuissoz PA (2013). Surface-length index: a novel index for rapid detection of right ventricles with abnormal ejection fraction using cardiac MRI. Eur Radiol.

[31] Gjesdal O, Almeida AL, Hopp E, Beitnes JO, Lunde K, Smith HJ, Lima JA (2014). Long axis strain by MRI and echocardiography in a postmyocardial infarct population. JMRI.

[32] Gjesdal O, Vartdal T, Hopp E, Lunde K, Brunvand H, Smith HJ, Edvardsen T (2009). Left ventricle longitudinal deformation assessment by mitral annulus displacement or global longitudinal strain in chronic ischemic heart disease: are they interchangeable?. J Am Soc Echocardiogr.

[33] Hung CL, Verma A, Uno H, Shin SH, Bourgoun M, Hassanein AH, McMurray JJ (2010). Longitudinal and circumferential strain rate, left ventricular remodeling, and prognosis after myocardial infarction. J Am Coll Cardiol.

